# Does the eversion technique have a lower early postoperative stroke rate than the conventional technique in carotid endarterectomy?

**DOI:** 10.1016/j.amsu.2022.104505

**Published:** 2022-08-28

**Authors:** Ahmed Abdel Rahim, Kareemaldin Elsamani, Ali Mahmoud Galal, Mohamed Ibrahim Hammoda, Devender Mittapalli

**Affiliations:** a University Hospitals Plymouth NHS Trust, UK; b University of Bahri, General Surgery Department, Kartoum, Sudan; c Aswan University Hospitals, Aswan University, Egypt; d Al Hussein University Hospitals, Al Azhar University, Egypt

**Keywords:** Carotid endarterectomy (CEA), Eversion carotid endarterectomy (ECEA), Conventional carotid endarterectomy (CCEA), Stroke, Transient Ischaemic attacks (TIA)

## Abstract

A best evidence topic has been constructed using a described protocol. The three-part question addressed was: In carotid surgery, Does the eversion technique (ECEA) has an early postoperative lower stroke rate, As compared to conventional carotid endarterectomy (CCEA)? The outcome assessed was the stroke rate in the early potoperative period (30 days) in the two techniques. The best evidence confirmed that there is no statistically significant difference between ECEA and CCEA regarding the early postoperative stroke incidence.

## Highlights

• Eversion technique and conventional endarterectomy are the main surgical endarterectomy techniques in carotid artery disease.
• There is no difference between the Eversion and conventional endarterectomy techniques in early postoperative stroke rates.
• Lack of randomised controlled trials that compare the two techniques.

## 1 Introduction

This BET was designed using a framework outlined by the International Journal of Surgery [1]. This format was used because a preliminary literature search suggested that the available evidence is insufficient to perform a meaningful meta-analysis. A BET provides evidence-based answers to common clinical questions using a systematic literature review. The outcome assessed was the stroke rate in the early potoperative period (30 days) in the two techniques. The best evidence confirmed that there is no statistically significant difference between ECEA and CCEA regarding the early postoperative stroke incidence.

## 2 Clinical scenario

While reviewing a 67-year-old man on day-1 post carotid endarterectomy using eversion technique, one of the junior doctors asked; Does the eversion technique has lower early postoperative stroke rates than the conventional endarterectomy?

## 3 Three parts question

• [In Carotid Surgery,]
• [Does the eversion technique has a lower early postoperative stroke rate ];
• [As compared to conventional endarterectomy technique]?

## 4 Search strategy

1. Embase 1974 to June 2021 using the OVID interface:

[Carotid artery disease]AND [Eversion endarterectomy or eversion technique] AND [Conventional endarterectomy OR Classic endarterectomy].

2. Medline using the PubMed interface:

[Carotid artery disease] AND [Eversion endarterectomy OR eversion technique] AND [Conventional endarterectomy OR Classic endarterectomy].

The results were limited to English articles and human studies.

• Inclusion criteria: all original articles review the postoperative stroke incidence among patients who underwent carotid endarterectomy using conventional or eversion techniques.
• Exclusion criteria: case reports, letters to the editor, conference abstracts and systematic reviews, and meta-analysis.

## 5 Search outcome

Using both search engines, we found a total of 172 articles. We excluded one hundred twenty-six articles because they were irrelevant based on the titles and or the abstracts. Forty-six full-text articles were screened and assessed for eligibility. We identified six papers to provide the best evidence to answer the question (see Table 1).

**Table 1:** Summary of search results.

Author/date of publication/journal/country	Study type and level of evidence	Patient group	Outcomes follow up	Key results	Additional comments
Cao et al., 2000, JVS, Italy [2].	Prospective randomized trial **Level 2**	Total of 1353 Patients *Group 1 ECEA: 678 patients *Group 2 CCEA: 675 patients Mean follow-up was 33 months	*Primary endpoint was: Early postoperative major strokes, death, and restenosis.	**Early postoperative stroke** **Early postoperative Stroke** Group 1 ECEA: 4 (0.60%) Group 2 CCEA: 2 (0.30%) *P value = 0.60. *Statistically Insignificant	*Late analysis of the EVEREST trial
Schneider J R et al., 2015, JVS, UK [3].	Retrospective observational study **Level 3**	Total of 19520 patients. * Group 1 ECEA: 2365 patients. *Group 2 CCEA: 17155 patients. * Mean Follow-up was ten years.	* Primary endpoint was: Early postoperative morbidity (CVA, MI, re-intervention), and mortality.	* Early postoperative stroke *Group 1 ECEA: 20 (0.8%) patients. *Group 2 CCEA: 152 (0.9) patients. *P value = 0.84 *Statistically Insignificant	*Large sampled size *Non-randomized retrospective analysis of prospectively collected data *1-year follow-up for about half of the patients
Djedovic M et al., 2017, Medical archives journal, Bosnia and Herzegovina [4].	Retrospective-Prospective study **Level 3**	*Total of 173 patients. *Group 1 ECEA: 90 patients *Group 2 CCEA: 83 patients *Follow-up was three years.	*Primary endpoint was: Early postoperative morbidity (CVA, MI) and mortality	**Early Postoperative stroke** Group 1 ECEA: 2 (2.2%) Group 2 CCEA: 4 (4.8%) *P value = 0.351. *Statistically Insignificant	*Conventional endarterectomy was with/without patching. * Specific outcomes monitoring (Perioperative and early postoperative period). .
Demirel S et al., 2012, Stroke-AHA journal, UK [5].	Retrospective observational study **Level 3**	*Total of 510 patients *Group 1 ECEA: 206 patients *Group 2 CCEA: 310 patients *Follow-up was two years	*Primary endpoint was: Early postoperative ipsilateral stroke or death	Early postoperative stroke *Group 1 ECEA: 19 (9%) patients *Group 2 CCEA: 9 (3%) patients *P value = 0.005. *Statistically significant	*Multicenter
Lee J H et al., 2014, Ann Surg Treat Res, Korea [6]	Prospective observational study **Level 2**	*Total of 120 patients. *Group 1 ECEA: 57 patients *Group 2 CCEA: 63 patients. * Follow-up was one year.	* Primary endpoint was: 1. Early(<30 days postoperative) outcomes: Stroke, MI, Cerebral palsy, CNI, mortality)	**Early postoperative stroke** Group 1 ECEA: 3 (5.3%) Group 2 CCEA: 4 (6.3%) *P value = 0.800. *Statistically Insignificant.	*Single center * One surgeon does all surgeries *Mid-term outcomes: 30 days- 1 year. *Late postoperative outcomes: > 1 year.
Yasa H et al., 2014, Neurochirurgie journal, Turkey [7].	Retrospective observational study **Level 3**	*Total of 380 patients. *Group 1 ECEA: 178 patients *Group 2 CCEA: 202 patients. * Mean follow-up was 26 months	* Primary endpoint was: Ipsilateral stroke or death * Secondary endpoint is MI, CNI, revision, and TIA.	**Early postoperative stroke** Group 1 ECEA: 1 (0.56%) Group 2 CCEA: 2 (0.99%) *P value = 0.762. *Statistically Insignificant	

## 6 Result

see Table 1

## 7 Discussion

Eversion and Conventional endarterectomy with primary closure or patch angioplasty are the most common surgical techniques of endarterectomy in the management of carotid artery disease [8].

The Conventional endarterectomy with primary closure is associated with higher restenosis rates, while using the patch is associated with higher infection rates. European guidelines of the European Society of Vascular Surgery and the Dutch society for vascular surgery consider CEA with patch angioplasty as the reference technique [9].

The main advantage of the ECEA is that there is no need for a patch that minimizes the operative time and risk of postoperative infection. However, the difficulty of inserting the shunt before removing the plaque and the high rates of postoperative hypertension due to transecting the carotid sinus nerve branches and loss of baroreceptors limit its use [9,10].

In this article, we reviewed the best studies which compared the ECEA to the CCEA, considering the early postoperative significant stroke rates.

Five of the six studies in our review are observational studies [[3], [4], [5], [6], [7]] and only one is a randomized trial [2]. Two studies had a large sample size of more than 1000 patients [2,3]. There was no significant difference in early postoperative stroke incidence, the exception being Demirel et al. study that reported a statistically significant high early postoperative stroke incidence in ECEA (9% versus 3%; p = 0.005). However, it appeared to offer higher protection from stroke between 30 days and two years post-operatively, as the 2-year risk of ipsilateral stroke in this study was significantly higher in the conventional CEA group (2.9% versus 0%; p = 0.017) [5].

This may because of the small sample size, and the fact that the choice of the endarterectomy echnique was left to the surgeons. Some surgeons or centers wouls prefer one technique over another. High or low surgeon related or centre-specific complications coiuldnot be ruled out in the analysis [5]. Our review was limited by the relatively weak level of evidence as there is only one randmised study and all the conventional endarterectomy comparisons included the primary closure and the patch angioplasty.

## Ethical approval

Ethical approval was not required.

## Source of funding

No source of funding

## Author contribution

Ahmed Abdel Rahim (AA): Conducted the literature search and wrote the paper. Rahi Karmarkar (RK): Assisted in the literature search and Writing of paper., Ali Mahmoud Galal (AG): Editing of writing, Mohamed Ibrahim Abd-El Rahman Hammoda (MH): Assisted in writing of paper., Devender Mittapalli (DM): Assisted in the literature search and writing of paper.

## Clinical bottom line

According to the above articles, the best evidence shows no significant difference between ECEA and CCEA regarding the early postoperative stroke incidence.

## Trail registry number

None.

## Garantor

Ahmed Abdel Rahim.

## Declaration of competing interest

No conflicts of interest.
